# 1246. Impact of Leveraging Clinical Decision Support Tools to Implement a Tiered Antimicrobial Stewardship Workflow at a Rural Academic Medical Center

**DOI:** 10.1093/ofid/ofad500.1086

**Published:** 2023-11-27

**Authors:** Catessa A Howard, Lauren Freeman, Meera Mehta, Derek Grimm, John A Guilfoose

**Affiliations:** West Virginia University Medicine, Morgantown, West Virginia; McLeod Health, Morgantown, West Virginia; WVU Medicine, Morgantown, West Virginia; WVU Medicine, Morgantown, West Virginia; West Virginia University, Morgantown, West Virginia

## Abstract

**Background:**

The Infectious Diseases Society of America and The Joint Commission recommend Antimicrobial Stewardship Programs (ASPs) incorporate preauthorization and/or prospective audit and feedback (PAF) as mechanisms for stewardship interventions. However, each institution must customize their ASP workflow. The ASP at West Virginia University Hospitals (WVUH), a rural, comprehensive academic medical center, was recently revamped by leveraging clinical decision support (CDS) tools via a tiered workflow in efforts to prioritize stewardship interventions for preauthorization and PAF.

**Methods:**

In March 2021 ASP pharmacists created a tiered workflow incorporating CDS tools from Theradoc® and Epic®. Review of patients identified on CDS alerts (Table 1), preauthorization, and PAF were conducted Monday through Friday 8:00 am to 5:00 pm by ASP pharmacist(s). Tiered workflow effectiveness was measured by internal and external benchmarking: antimicrobial days of therapy (DOT)/1000 days present, antimicrobial spend, and Standardized Antimicrobial Administration Ratio (SAAR). Interventions were documented within the electronic medical record and tracked via a Quality Assurance and Performance Improvement (QAPI) format. Post implementation data from March 2021 to March 2023 was analyzed and compared to baseline 2019 data.

**Figure:**
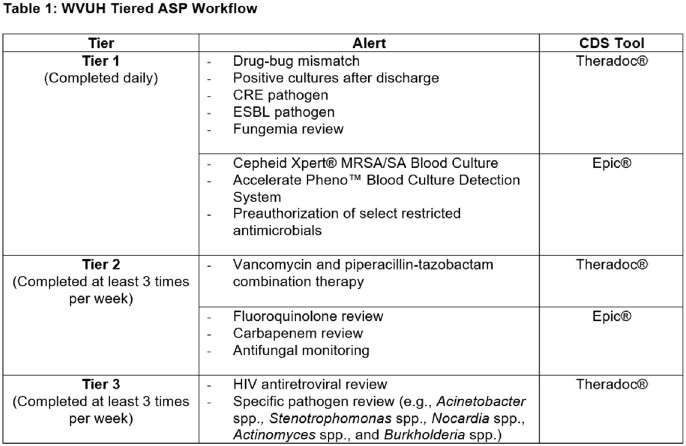

CRE: Carbapenem-resistant Enterobacterales; ESBL: Extended Spectrum Beta-Lactamase; HIV: Human Immunodeficiency Virus

**Results:**

The following outcomes were observed within the post implementation timeframe. Monthly average DOT/1000 days present decreased ≥ 20% for the following antimicrobials: vancomycin (27%), ceftaroline (53%), cefepime (20%), ertapenem (59%), aztreonam (68%), tigecycline (23%), fosfomycin (80%), micafungin (27%), ceftazidime-avibactam (40%). Monthly average total antimicrobial SAAR decreased by 11% from 1.216 to 1.074. Additionally, monthly average antimicrobial spend/patient day decreased by 36% from $26.47 to $16.95. QAPI data demonstrated a 74% intervention acceptance rate including correction of 344 drug-bug matches.

**Conclusion:**

Implementation of a tiered ASP workflow effectively identified patients for interventions, decreased antimicrobial utilization, and demonstrated antimicrobial cost containment. Acceptance of stewardship interventions was favorable. This tiered approach may serve as a model for other ASPs.

**Disclosures:**

**All Authors**: No reported disclosures

